# A315 A QUALITY ASSESSMENT OF THE ADEQUACY OF FLUID REPLACEMENT THERAPY FOR PATIENTS DIAGNOSED WITH ACUTE PANCREATITIS

**DOI:** 10.1093/jcag/gwad061.315

**Published:** 2024-02-14

**Authors:** K Patel, M Niazi, S Khan, P Mathura, J Zhang, G Sandha

**Affiliations:** Medicine, University of Alberta Faculty of Medicine & Dentistry, Edmonton, AB, Canada; Royal Alexandra Hospital, Edmonton, AB, Canada; Medicine, University of Alberta Faculty of Medicine & Dentistry, Edmonton, AB, Canada; Internal Medicine, University of Alberta, Edmonton, AB, Canada; Alberta Health Services, Edmonton, AB, Canada; Medicine, University of Alberta Faculty of Medicine & Dentistry, Edmonton, AB, Canada

## Abstract

**Background:**

For acute pancreatitis (AP), aggressive fluid resuscitation therapy (FRT) is the cornerstone for initial management. However, results from the recent WATERFALL study show a higher incidence of fluid overload with aggressive FRT (bolus of 20 ml/kg followed by infusion of 3 ml/kg/hr of Ringer’s lactate [RL]) compared with moderate FRT (infusion of 1.5 ml/kg/hr RL preceded by a bolus of 10 ml/kg only in those with hypovolemia). Given this evidence, we conducted a study to assess whether patients with AP received appropriate fluid therapy and monitoring at our institution.

**Aims:**

To assess whether fluid replacement therapy in patients with AP was aligned with updated clinical practice at our centre.

**Methods:**

We completed a chart audit of all adult (≥ 18 years old) patients who presented to the ED from April 1, 2021 through March 31, 2022 and were diagnosed with AP. Patients were identified based on ICD-10 codes for a primary admission or discharge diagnosis of AP. Data variables collected included demographic information, cause of AP, comorbidities, laboratory tests, type and volume of fluids replaced, urine output, complications, and hospital length of stay (HLOS). Severity of AP was categorized as mild, moderate, or severe based on the revised Atlanta classification of AP. Descriptive statistics were completed.

**Results:**

A total of 250 patients (121 F, 129 M), mean age 53±19 years (range 19-96 years) were diagnosed with AP. Body weight was not available for 4 patients and thus excluded from this analysis. FRT variables stratified for the severity of AP are below in Table 1.

**Conclusions:**

Our quality assessment has identified a knowledge-to-practice gap in the use of FRT for patients with AP. To assist physicians in the decision-making process, we have developed an algorithm to improve FRT in AP. This will be implemented via an order set to standardize the amount of FRT and to improve monitoring of urine output. This algorithm will be tested using an improvement science approach in collaboration with healthcare professionals.

Table 1. Summary of fluid management in AP patients as stratified by severity of disease.

**Funding Agencies:**

None

**Table:** 

	MILD AP	MODERATE AP	SEVERE AP	TOTAL
PATIENTS, n (%)	195 (80)	38 (5)	13 (5)	246
COMPLICATIONS, n (%)	15 (7)	4 (11)	7 (54)	26 (11)
DEATHS, n (%)	0 (0)	0 (0)	2 (15)	2 (1)
AVERAGE HLOS, days	5	7	28	
FLUID BOLUS RECEIVED, n (%) VOLUME, ml/kg mean±SD	149 (76) 15±2	33 (87) 17±5	11 (85) 22±5	193 (78) 16±2
MAINTENANCE FRT, n (%) ampersand:003C1.5ml/kg/hr 1.5-3ml/kg/hr ampersand:003E3 ml/kg/hr	90 (46) 102 (52) 3 (2)	12 (32) 26 (68) 0 (0)	3 (23) 7 (54) 3 (23)	105 (43) 135 (55) 6 (2)
URINE OUTPUT RECORDED, n (%) VOLUME, ml/24 hrs mean±SD	13 (7) 435±221	1 (3) 30	5 (39) 939±639	19/246 (7) 546±244

